# Cervical cancer screening programme attendance and compliance predictors regarding Colombia’s Amazon region

**DOI:** 10.1371/journal.pone.0262069

**Published:** 2022-01-25

**Authors:** Alejandra González, Ricardo Sánchez, Milena Camargo, Sara Cecilia Soto-De León, Luisa Del Río-Ospina, Luis Hernando Mora, Edwin Ramírez, Anny Alejandra Rodríguez, Paula Hurtado, Manuel Elkin Patarroyo, Manuel Alfonso Patarroyo

**Affiliations:** 1 Molecular Biology and Immunology Department, Fundación Instituto de Inmunología de Colombia (FIDIC), Bogotá, Colombia; 2 Faculty of Medicine, Universidad Nacional de Colombia, Bogotá, Colombia; 3 Animal Science Faculty, Universidad de Ciencias Aplicadas y Ambientales (U.D.C.A), Bogotá, Colombia; 4 Ministerio de Relaciones Exteriores, Bogotá, Colombia; 5 Health Sciences Division, Main Campus, Universidad Santo Tomás, Bogotá, Colombia; University of Central Florida, UNITED STATES

## Abstract

**Background:**

Cervical cancer (CC) promotion and prevention (P&P) programmes’ challenge lies in guaranteeing that follow-up strategies have a real impact on reducing CC-related mortality rates. CC P&P programme compliance and coverage rates are relevant indicators for evaluating their success and good performance; however, such indicators’ frequency rates are considerably lower among women living in rural and border areas. This study was aimed at identifying factors associated with CC screening programme attendance for women living in Colombia’s Amazon region.

**Methods:**

This study (qualitative and quantitative phases) was carried out between September 2015 and November 2016; women residing in the border towns of Leticia and Puerto Nariño participated in it. The first phase (qualitative) involved interviews and focus group discussions; this led to establishing factors related to CC P&P programme attendance which were used in the quantitative phase for designing a survey for determining the strength of association in a logistic regression model. The terms attendance and compliance were considered to apply to women who had followed the 1–1–3 scheme throughout their lives, i.e. a cytology examination every 3 years after receiving two consecutive negative annual cytology results.

**Results:**

Inclusion criteria were met by 309 women (≥18-year-olds having an active sexual life, having resided in the target community for at least one year); 15.2% had suitable P&P programme follow-up. Screening programme attendance was positively associated with first intercourse after becoming 20 years-old (aOR: 3.87; 1.03–9.50 95%CI; *p* = 0.045), frequent contraceptive use (aOR: 3.11; 1.16–8.33 95%CI; *p* = 0.023), awareness of the age to participate in P&P programmes (aOR: 2.69; 1.08–6.68 95%CI; *p* = 0.032), awareness of cytology’s usefulness in identifying cervical abnormalities (aOR: 2.43; 1.02–5.77 95%CI; *p* = 0.043) and considering cytology important (aOR: 2.64; 1.12–6.19 95%CI; *p* = 0.025). Women living in rural areas had a lower probability (aOR 0.43: 0.24–0.79 95%CI; *p* = 0.006) of adhering to CC P&P programmes.

**Conclusions:**

This study’s findings suggested the need for including novel strategies in screening programmes which will promote CC P&P activities going beyond hospital outpatient attendance to reach the most remote or widely scattered communities, having the same guarantees regarding access, opportunity and quality. Including education-related activities and stimulating the population’s awareness regarding knowledge about CC prevention could be one of the main tools for furthering the impact of attendance at and compliance with P&P programmes.

## Introduction

Many Latin American cervical cancer (CC) screening programmes have been functioning for more than 40 years; their essential role lies in introducing programmes for the prevention and early detection of CC [1]. This goal is still far from being achieved; CC is the second most commonly diagnosed cancer in Latin America and the Caribbean, mainly among women of childbearing age [2]. CC occupies third place amongst the types of cancer affecting women in Colombia; 3,853 CC cases are diagnosed annually (12.7 per 100,000 women per year) and 1,775 cases of CC-related mortality are reported every year (5.7 per 100,000 women per year) [3].

Persistent human papillomavirus (HPV) infection contributes to CC development; nevertheless, viral presence does not necessarily lead to full-blown disease [4]. Only a small percentage of HPV infections become persistent and only a few persistent HPV infections might progress to CC after several decades [5]. CC could thus be preventable if HPV infection and pre-cancerous lesions are detected early via cervical screening (i.e. promotion and prevention; P&P) programmes [6].

Developing countries face many challenges when trying to ensure suitable CC detection coverage and running effective cervical cytology-based programmes on [7]. However, coverage is not the only indicator which should be considered; monitoring is also important as monitoring indicators will reveal programme success [8, 9]. Many challenges are involved in cervical screening programme compliance with attendance at the times proposed within the cytology schemes and claiming result; this becomes exacerbated for women living in rural regions having high sociodemographic and environmental diversity [10–12]; this explains why follow-up levels are lower for populations living in rural and frontier areas [9, 13, 14].

The Colombian Amazon is one of the country’s regions having the greatest ethnic and ecosystem variety [15] as it borders Peru and Brazil and has high population mobility owing to its rivers’ navigability [16]. Entering and travelling within this territory is difficult; there is little access to medical care and close to 40% of the population (mainly those living in dispersed rural and/or populated urban centres) live in poverty. Households are characterised by unemployment, informal employment, illiteracy, critical overcrowding, their homes being built with inadequate construction materials, inadequate disposal of excreta and limited access to basic sanitation services [17, 18]. The Colombian Amazon region has high CC-related mortality rates compared to other regions of Colombia; the Amazon department’s 2019 mortality rate was 26.3 per 100,000 inhabitants compared to the national average of 9.7 per 100,000 inhabitants [19].

Colombian CC screening regulations state that all women aged 25 to 69 years who are affiliated to Colombia’s healthcare system should be able to have unrestricted access to free conventional cervical cytology taking and reading. This also extends to minors/underage females having access to the examination once they have initiated sexual activity. Progress has been made towards including new methodologies, such as liquid-based cytology, automation-assisted reading, and introducing complementary testing, such as the HPV DNA test [20, 21].

Colombia’s healthcare system uses a 1-1-3 cytology scheme [20]; the regulations state that if a woman has a negative result in two consecutive cytologies (spaced one year apart), then cytology must be performed every three years from then on, as long as the result remains negative. This scheme has been shown to have a good incremental cost-effectiveness ratio (ICER) [20, 21].

No studies aimed at establishing factors associated with P&P programme follow-up failure have been carried out in Colombia. Ascertaining the related data will facilitate strengthening actions focused on designing and improving pertinent attendance, compliance and adherence strategies that mainly affect women living in remote regions and those having difficult access to healthcare [22]. This study’s objectives were to identify factors involved in attendance at and compliance with a 1–1–3 P&P scheme (through in-depth interviews with health personnel and focus groups with women from the local communities) and determine the frequency of and factors involved in attendance at and compliance with CC detection programmes regarding women living in urban and rural Amazon areas. This study’s follow-up population was defined as the women who fulfilled Colombia’s technical regulations establishing 1–1–3 scheme periodicity for cervical cytology. Regular attendance at and compliance with the scheme (yes/no) was determined by comparing each surveyed woman’s age, age at first sexual intercourse versus lifetime amount of cytology examinations versus the date of last cytology versus age at the time of the survey. This information led to establishing a window of 5-year intervals between the age upon starting sexual relations and age when the survey was taken. Programme compliance was established if a woman had received three cytology exams every 5 years.

## Materials and methods

### Study design and participants

Mixed methods were used in this study (qualitative and quantitative research); inclusion criteria for the qualitative and quantitative components were women aged 18 to 69 years-old who were sexually active and who had been living in the municipalities of Leticia and Puerto Nariño in Colombia’s Amazon region for at least 1 year. Exclusion criteria stated that women could not be aged less than 18 years-old or lacked a sexual partner at the time of the study. This population is of great interest owing to the region’s characteristics, which limits access among its scattered communities to P&P programmes in terms of CC diagnosis and where healthcare is often ignored.

The study was carried out in two phases; the first was a qualitative study (September 2015 to March 2016) comprising in-depth interviews and focus group discussions (FGDs). Qualitative analysis and its results were used as input in the second part of the study (the quantitative part was carried out from April to November 2016), leading to a survey for evaluating factors associated with the women’s attendance at and compliance with a CC P&P programme.

Three researchers used ATLAS.ti qualitative analytical software v.7.0 (Scientific Software Development GmbH, Berlin, Germany) to triangulate the information and avoid bias. The S1 File provides the research team’s methodological guidelines [23, 24].

Each researcher carried out a dedicated and methodical reading of each transcript to identify the units of analysis (codes) referring to experiences, representations, perceptions and images related to the screening programme. The codes identified by each researcher were compared and the recurring ones suggested the dimensions to be created for analysis. Five dimensions were defined: socioeconomic and demographic, risk factors, screening programme accessibility, perceived cervical screening programme quality and participants’ level of knowledge regarding CC prevention mechanisms.

### First phase: Qualitative study

Indigenous and non-indigenous women aged 18 to 69 years-old, having lived for at least 1 year in the selected communities and leading an active sexual life (i.e. having at least one sexual partner at the time the FGDs were held) participated in the FGDs (Table 1). Both the area’s urban (more than 100 inhab/km^2^) and rural populations (less than 100 inhab/km^2^) were included [25]; demographic data provided by the departmental secretariat of health was used to select the rural settlements. The most densely populated communities (having more than 100 individuals) along with urban settlements having P&P programme coverage were selected.

**Table 1:** Communities selected for the focus group discussions.

**Leticia**	**Area**	**Community/barrio**	**# Women included**	**Puerto Nariño**	**Area**	**Community/barrio**	**# Women included**
	Rural	San Sebastian de los Lagos	4		Rural	Veinte de Julio	8
						Puerto Esperanza	13
		La Milagrosa	5			San Juan de Atacuari	11
		San Antonio de los Lagos	10			Siete de Agosto	3
						Naranjales	12
		Isla de la Fantasía	6			San Pedro de Tipísca	8
	Urban^a^	El Águila neighbourhood	6			Doce de Octubre	8
		IANE neighbourhood	4			San Francisco	5
		Castañal neighbourhood	4			San Juan del Socó	4
		José María Hernández neighbourhood	8		Urban	Los Baos neighbourhood	8

^a^ Two focus groups were held in Leticia’s urban area, the first in El Águila and IANE neighbourhoods and the second in the Castañal and José María Hernández neighbourhoods.

Community leaders and organisations (Cabildos Indígenas del Trapecio Amazónico, Asociación Zonal de Consejo de Autoridades Indígenas de Tradición Autóctona) along with public health network nursing assistants and “curacas” (political and administrative chiefs who are considered the leaders of and spokesmen for the local indigenous people) from the communities of interest collaborated in issuing the invitation for women to participate in the FGDs. The chain of information then required that the study objectives, FGD topics and criteria for selecting the women had been explained to the aforementioned individuals/leaders. They then transmitted this information to those in their zones of influence and held meetings within their communities concerning interest in study participation. Communities where the FGDs were held expressed their interest in voluntarily participating in the study via their traditional leaders and/or community organisations.

An invitation to participate was designed to guarantee that the participant selection criteria were fulfilled and that the methodology to be used would respect the indigenous and communal organisations’ conventions. The FGDs were to last around 90 minutes and would have a coordinator and two assistants. All meetings were digitally recorded for later transcription.

A written invitation to participate in the in-depth interviews was issued to all personnel (general practitioners, gynaecologists, nurses, nursing assistants and administrative staff) having experience or knowledge regarding introducing or executing local P&P programmes and who were working in the region’s public and/or private institutions: Hospital San Rafael de Leticia, Hospital Local de Puerto Nariño, the Amazonas Cancer League, the Mallamas indigenous health service-promoting entity, departmental public health and epidemiology surveillance teams and municipal and departmental secretariats of health. All (n = 17) functionaries who voluntarily agreed to participate signed an informed consent form and were interviewed. All 60-minute interviews were digitally recorded and transcribed. There was no structured questionnaire for the interviews; these were rather of an open-ended nature having some questions (S1 Table in S1 File) and conversations related to the dimensions used in the FGDs.

Each transcript was read by three researchers using ATLAS.ti qualitative analytical software v.7.0 (Scientific Software Development GmbH, Berlin, Germany) to triangulate the information and avoid bias. The S1 File provides the research team’s methodological guidelines [24].

Each researcher carried out a dedicated and methodical reading of each transcript to identify the units of analysis (codes) referring to experiences, representations, perceptions and images related to the screening programme. The codes identified by each researcher were compared and recurring ones suggested the dimensions to be created for analysis. Five dimensions were defined: socioeconomic and demographic, risk factors, screening programme accessibility, perceived cervical screening programme quality and participants’ level of knowledge regarding CC prevention mechanisms. Such dimensions have been described as social determinants of health (SDH) [12, 26, 27] and progress has been made regarding SDH-related knowledge concerning Colombian populations; nevertheless, such information regarding populations living in Colombia’s Amazon is still limited.

All analysis instruments (FGDs and in-depth interviews) were constructed from elements taken from the PRECEDE-PROCEED rural health planning model (predisposing, reinforcing and enabling causes in educational diagnosis and evaluation) [23, 24] and written in Spanish as the target population understood and spoke this language. Table 2 outlines the topics dealt with during the in-depth interviews and FGDs.

**Table 2:** Topics dealt with during the in-depth interviews and focus group discussions.

Main topic	Secondary topics
Socioeconomic	The municipality’s main economic activities, settlement patterns, social organisation, expectations, wishes, needs. P&P programme users’ perception of quality of life and basic unsatisfied needs; health service providers’ extramural activities.
Cultural–behavioural	Cultural characterisation, the most relevant cultural practices, cultural risk factors considered most relevant, relevant historical events, migrations, potentialities, resistance and ability to adapt to change, social support networks. Personal knowledge/awareness of the municipality’s ethnic diversity. Testimonies regarding previous experience for improving women’s attendance at and compliance with P&P programmes (local strategies). Previous experience of working with an indigenous population and health P&P programmes.
Epidemiological	Health profile, demography, morbidity, mortality, level of knowledge regarding parts of the body, location of the cervix, knowledge concerning CC and HPV.
Environmental	The municipality’s geographical characteristics and those of the users attending P&P programmes, limitations regarding access, knowledge of the itinerary to access health services which users must be aware of, resources used/needed for accessing health services.
Political/policy-based administrative	Assurance, relationship with insurers and surveillance and control bodies, detailed description of functions, description of the health service-providing network, characterising health service usage, testimonies regarding access to CC control programmes. Description of how P&P activities are developed. Users’ degree of satisfaction with the health service provider. Personal perception of P&P programme functioning. Level of personal knowledge and perception regarding current Colombian regulations for CC control, knowledge/awareness of rights concerning healthcare. Level of knowledge and perception of the quality of screening strategies. Managing epidemiological and public health information.

### Second phase: Quantitative study

Analysing interview and FGD transcript topics and content led to defining the factors to be included in the survey. The study’s first phase ended by identifying the factors influencing the women’s attendance at and compliance with CC P&P programmes; this led to the second phase involving a quantitative study for compiling, analysing, designing and using this instrument (survey) with the target population, that took place between April and November 2016. Survey results were used for measuring the strength of association (odds ratio; OR) for factors related to attendance at and compliance with CC screening programmes in the region.

The research team (consisting of an anthropologist, psychologist, doctor, statistician, biologist, bacteriologist, and a systems engineer) collectively reviewed each dimension, defined the factors for each dimension and designed the survey questions which led to obtaining the preliminary version.

**Fig 1:**
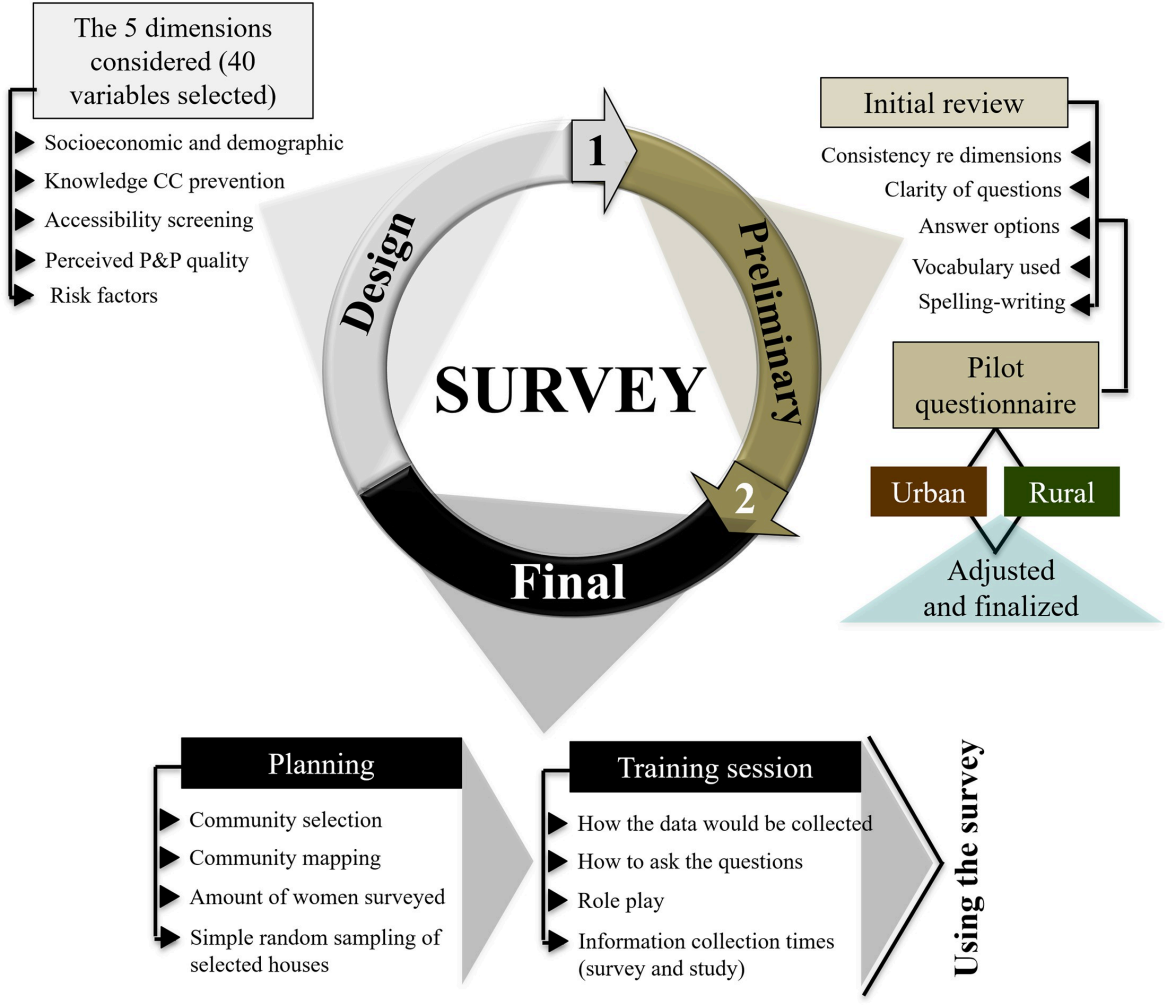
Flowchart explaining survey design, planning and its preliminary and final versions.

The survey’s preliminary version was evaluated by an external researcher (an expert in P&P programmes) and reviewed by the Amazon Department of Health personnel responsible for managing its sexual and reproductive health programmes. The survey questionnaire was then tested in two communities (urban and rural) by a researcher (observer) and a healthcare agent (interviewer), adjusted and finalised (Fig 1).

The survey (close-ended questions) took the form of face-to-face interviews; S2 and S3 Tables in S1 File list all the factors included in the survey. Public health technicians who had received training in correctly completing the survey administered it during October and November 2016 in four urban and nine rural communities regularly visited by the departmental secretariat of health’s healthcare promoters (Table 3).

**Table 3:** Communities selected for the survey.

**Leticia**	**Area**	**Community/Barrio**	**# of houses**	**Puerto Nariño**	**Area**	**Community/Barrio**	**# of houses**
	Rural	San Martin	77		Rural	Puerto Esperanza	80
		Macedonia	111				
		Kilómetro 18	109			San Francisco	88
		Ronda	90				
		Isla de la Fantasía	98			San Juan del Socó	157
		Arara	165				
	Urban	Nazareth	162				
		Tauchi neighbourhood	127		Urban	Los Baos neighbourhood	102
		Victoria Regia neighbourhood	118				

The selected communities were mapped to locate the communities’ streets, houses, rivers and boundaries. The houses were then numbered and the number of houses to be surveyed was estimated using proportionality, taking the total number of houses per community into account, to calculate the total sample. Simple random sampling was used to select the houses for the survey.

The women surveyed had sufficient physical and cognitive abilities to be able to answer the survey questions. If a household included more than one woman who fulfilled the survey inclusion criteria, then lots were drawn to choose only one woman to participate. Houses where an adult did not wish to take part in the survey or where no one was over 18 years old were not surveyed and the next randomised house was then approached. FGDs were held in five of the communities involved in the surveys; the women living in these communities completed the survey if their houses had been selected.

Sociodemographic characteristics and clinical information were compiled during the first part of the survey; this included participants’ age, ethnicity, educational level, marital status, occupation, access to water, sewerage and/or gas (i.e. public services), monthly income, amount of sexual partners and age at first sexual intercourse. The second part of the questionnaire dealt with P&P programme accessibility and quality factors, such as locations of the houses, means of transport for reaching screening programme sites, the amount of money needed for getting to the sites and women’s perception of the time until they received cytology results. The third part asked about the women’s level of knowledge regarding tools for CC prevention, cytology, HPV and community participation. Factors such as awareness about the cytology examination, age at first cytology, preparation for the cytology examination and knowledge concerning HPV were considered.

Information collected from 10% of the surveys was corroborated in phone calls to participants to minimise bias as health service promoters actually conducted the survey among the target populations and the questions were related to topics addressing sexual behaviour and P&P programme accessibility and quality. A person who was not involved in the study verified such data.

Two researchers using a Microsoft Access database independently digitised the survey responses; both versions were then compared to correct any errors.

### Measurement and statistical analysis

FGDs were analysed by taking the occurrence of redundant information into account, i.e. the point at which repeated information or topics were observed and no new information was found, the same codes recurring [24, 28]. The number of interviews was determined by the number of functionaries who accepted the invitation to voluntarily participate in the study. EpiData (v.2) was used to calculate survey sample size, assuming that the factors having the greatest heterogeneity would occur in the population at a rate of at least 50% (i.e. factor frequency determination, 0.05 estimator precision and 95% confidence interval (95%CI)). Sample size was 385 women; the number of women surveyed per community was adjusted taking proportional assignment into account as sample size depended on the number of houses per community. EpiData (v.3.1) software was used to handle tabulated data, i.e. carrying out all calculations.

Women were excluded from analysis when CC P&P programme attendance and compliance could not be established because they had not answered questions regarding their age, age upon beginning sexual relations, lifetime number of cytology examinations and date of last cytology. Stata 12.0 statistical package was used for analysing the data (StataCorp LLC, College Station, TX, USA).

Descriptive statistics were used to analyse the clinical information. Age was reported together with mean and standard deviation (SD); categorical factors were expressed in terms of frequency with 95%CIs.

Ordinal logistic regression models were used for estimating aORs, considering participants’ attendance at and compliance with cervical cytology screening-based P&P programmes as dependent variable. This type of regression was used it enabled outcome having more than two uniformly distributed categories having a natural order to be analysed (polyatomic ordinal response regarding the nature of the data).

Programme attendance and compliance were categorised into three levels: total non-attendance (zero) included women who had never attended a CC P&P programme, attendance at least once (one) women who had had at least one cytology during their lifetimes but did not comply with the 1-1-3 scheme and attendance and compliance (two) women who had had a cytology and complied with the 1-1-3 scheme at some time during their lives. Socioeconomic and demographic factors, risk factors, accessibility, perceived P&P programme quality and awareness of CC prevention were the independent variables (S2 and S3 Tables in S1 File).

Variance inflation factor (VIF), tolerance and eigenvalues were used for establishing multicollinearity diagnostics [29, 30]. Power analysis was calculated using two-tailed ordinal outcome comparison tests, assuming odds proportionality in a logistic model (S4 and S5 Tables in S1 File). R software was used for these calculations, using the Hmisc library popower function (power and sample size calculations for ordinal responses) which uses Whitehead’s algorithm [31].

### Ethical considerations

The study’s scope was explained to the women who were included and who agreed to participate by signing an informed consent form. Participants were assigned a unique, consecutive number once they had entered the study and their personal data (name and citizen identification number) were anonymised. All data provided by the women was only used for research purposes; they were assured that the data would not be made available to third parties. All procedures performed in this study were approved and supervised by the Universidad del Rosario’s (Bogotá, Colombia) School of Medicine and Health Sciences Research Ethics Committee, CEI-ABN026-000160 (May 12, 2014).

## Results

The 17 functionaries participating in the study described (in terms of their professional experience) lack of participation in screening programmes among women and the region’s cultural diversity. The functionaries also identified political–administrative and financial elements having an impact on early detection programme functioning, level of knowledge regarding current Colombian regulations for controlling CC, and administrators and health service providers’ willingness (or lack thereof) to integrate their screening strategies into the cultural specificities of the female population.

FGD information saturation (i.e. codes beginning to recur) began after FGD 16; 127 women participated in the FGD in 6 groups in Leticia (2 in an urban area and 4 in a rural area) and 10 in Puerto Nariño (1 in an urban area and 9 in a rural area) (Table 1). The FGD provided a non-confrontational space for dialogue, enabling the women to explore different visions and ways of thinking and feeling about cytology, their perceptions related to health provider service quality, opportunity and access, the relationship between health service personnel and patients, the setting’s characteristics, and a detailed description of how they travelled to health centres/posts in their area. Reflection about their bodies and self-care emerged from the FGDs, helping participants to understand how each perceived health, disease, and healthcare.

Coding involved reading an average of 1,112 fragments of text in which approximately 170 codes were identified using Atlas.ti 5.2 (Archive for Technology, Lifeworld and Everyday Language.text interpretation–Berlin Scientific Software Development) software, producing a list ordered from greater to lesser frequency of occurrence. This list led to identifying that the most frequently occurring codes referring to the timely delivery of cytology results, a lack of knowledge regarding how cytology works, few extramural activities offered by health service-providing entities, and a lack of information and geographic limitations regarding access to health centres/posts providing cytology services.

We then analysed correlation between factors using social network analysis to facilitate the identification of grouping patterns and to identify general factors containing more specific ones. The survey was then designed based on a final list of 40 factors covering the five dimensions of analysis.

**Fig 2:**
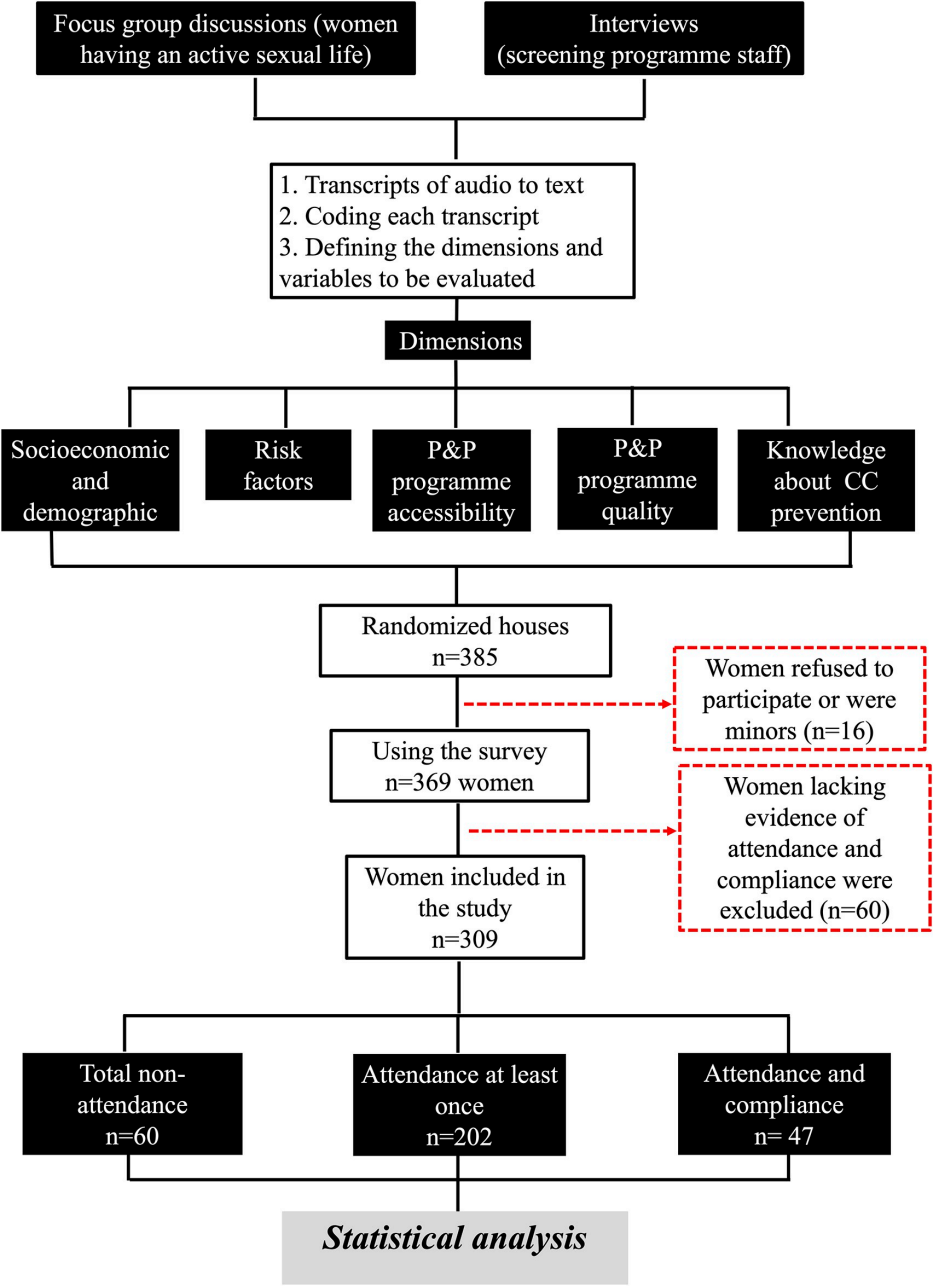
Flowchart explaining the design and the women included in the study.

Survey data collection involved randomising 385 houses; data could not be obtained in 16 of these because the women refused to participate or because there were no women over 18 years old in the household at the time of the visit. Thus, we included 369 women living in the target area (Table 3); a further 60 women were excluded because follow-up information was not identified (attendance and compliance could not be determined), thereby excluding them from the statistical analysis (Fig 2), leaving a total of 309 women in the final analysis. We found that 19.4% (n = 60) of the women had never participated in a cervical screening programme for early detection of CC whereas 80.6% (n = 249) had undergone a cytology examination at least once in their lives; among this last group, 15.2% of women (n = 47) satisfactorily complied with scheduled times for cytology examinations in the follow-up scheme.

It was found that 61.2% of the population (n = 189) lived in rural areas and 74.1% (n = 229) were indigenous; Ticuna was the most represented ethnic group in the target population. Regarding sexual behaviour, mean age at first sexual intercourse was 16.2 (SD = 2.4) years old and 38.5% (n = 119) of women reported that they did not use contraceptive methods. Most women (13.3%) who stated that they had had no formal education were in the Total non- attendance group and only 12% (30/249) of the women who had attended CC screening (Attendance at least once or attendance and compliance) had received the result of their last cytology. Table 4 lists other characteristics of the study population.

**Table 4:** Sociodemographic description of the study population.

Demographic	Total non- attendance	Partial attendance	Attendance and compliance	Total
	(*n* = 60)	(*n* = 202)	(*n* = 47)	
**Age (mean [range])**	40.3 [18–67]	37.3 [18–69]	36.1 [19–68]	**37.7 [18–69]**
	SD = 12.6	SD = 10.5	SD = 12.5	**SD = 11.3**
**Age on first intercourse (years) (mean [range])**	16.7 [13–23]	15.9 [11–27]	17.1 [13–23]	**16.2 [11–27]**
	SD = 2.4	SD = 2.3	SD = 2.3	**SD = 2.4**
	***n (%)***	***n (%)***	***n (%)***	***n***
**Location of dwelling**				
Urban	33 (55.0)	68 (33.7)	19 (40.4)	**120**
Rural	27 (45.0)	134 (66.3)	28 (59.6)	**189**
**Educational level**				
None	8 (13.3)	15 (7.4)	0 (0.0)	**23**
Primary/secondary	41 (68.3)	170 (84.2)	39 (82.9)	**250**
Technical/professional	11 (18.3)	17 (8.4)	8 (17.0)	**36**
**Occupation**				
Housewife	21 (35.0)	86 (42.6)	14 (29.8)	**121**
Agriculture/small farm/plot worker	16 (26.7)	61 (30.2)	15 (31.9)	**92**
Businesswoman	4 (6.7)	12 (5.9)	3 (6.4)	**19**
Other	19 (31.7)	43 (21.3)	15 (31.9)	**77**
**Ethnicity**				
Ticuna indigenous	29 (48.3)	125 (61.9)	26 (55.3)	**180**
Mestizo	22 (36.7)	39 (19.3)	17 (36.2)	**78**
Other indigenous group ^a^	9 (15.0)	36 (17.8)	4 (8.5)	**49**
Afro descendent	0--	1 (0.5)	1 (2.1)	**2**
**Contraceptive method**				
None	18 (30.0)	86 (42.6)	15 (31.9)	**119**
Hormonal	17 (28.3)	61 (30.2)	15 (31.9)	**93**
Tubal ligation	13 (21.7)	35 (17.3)	9 (19.1)	**57**
Other method ^b^	12 (20.0)	19 (9.4)	9 (19.1)	**40**
**Pregnancies**				
None	2 (3.3)	2 (1.0)	0 (0.0)	**4**
1–2	17 (28.3)	42 (20.8)	23 (48.9)	**82**
≥3	41 (68.3)	157 (77.7)	25 (53.2)	**223**
**Abortions**				
No	50 (83.3)	148 (73.3)	35 (74.5)	**233**
Yes	10 (16.7)	53 (26.2)	13 (27.7)	**76**
**Lifetime amount of sexual partners**				
1	38 (63.3)	108 (53.5)	25 (53.2)	**171**
2–3	13 (21.7)	40 (19.8)	14 (29.8)	**67**
>3	9 (15.0)	54 (26.7)	8 (17.0)	**71**
**Current smoker**				
No	49 (81.7)	184 (91.1)	45 (95.7)	**278**
Yes	11 (18.3)	18 (8.9)	2 (4.3)	**31**
**Contraceptive use**				
Never	44 (73.3)	150 (74.3)	25 (53.2)	**219**
Occasionally	9 (15.0)	35 (17.3)	15 (31.9)	**59**
Always	7 (11.7)	17 (8.4)	7 (14.9)	**31**
**Did you receive the result of your last cytology?**				
No	0 --	175 (86.6)	44 (93.6)	**219**
Yes	0 --	27 (13.4)	3 (6.4)	**30**

^a^ Other indigenous group included Uitoto, Yaguas and Cocamas.

^b^ Other method included medicinal plants and natural contraceptives.

The factors were evaluated regarding the five aforementioned dimensions (materials and methods section) for estimating associations in the study population. Univariate analysis revealed positive associations regarding attendance at and compliance with cervical cytology screening for five factors, including always using contraception (OR: 2.09; 95%CI1.11–3.94) and awareness of the usefulness of cytology in identifying cervical abnormalities (OR: 2.13; 95%CI1.30–3.14). By contrast, a negative association was found for three factors, including being a current smoker (OR: 0.41; 95%CI0.18–0.94). S2 and S3 Tables in S1 File show other associations from the results of univariate analysis; these factors were no longer significant after adjustment.

Following OR adjustment (aOR), some factors continued to be positively associated in multivariate analysis, i.e. constant condom/contraceptive use (aOR: 3.11; 1.16–8.33 95%CI; power: 0.998) and being over 20 years old upon becoming sexually active (aOR: 3.87; 1.03–9.50 95%CI; power: 0.999) (Table 5). Other associations were found, such as awareness of the usefulness of cytology in identifying cervical abnormalities (aOR: 2.43; 1.02–5.77 95%CI; power: 0.966), recognising the importance of cytology (aOR: 2.64; 1.12–6.19 95%CI; power: 0.985) and knowing when to begin receiving cytology examinations (aOR: 2.69; 1.08–6.68 95%CI; power: 0.988) (Table 6). There was a lower probability (aOR 0.43: 0.24–0.79 95%CI; power: 0.949) of women living in rural areas having access to CC P&P programme follow-up (Table 5). Multicollinearity diagnostics revealed VIF <2, tolerance >0.21, and eigenvalues >0.14, indicating no collinearity between the factors included in the model.

**Table 5:** Univariate and multivariate analysis of socioeconomic and demographic factors and risk factors associated with attendance and compliance (n = 309 women).

Demographics	Total non- attendance	Attendance at least once	Attendance and compliance	Univariate analysis		*p*-value	Multivariate analysis		*p*-value
	*n* (%)	*n* (%)	*n* (%)	OR	95%CI		OR^a^	95%CI	
**Location of dwelling**									
Urban	33 (55.0)	68 (33.7)	19 (40.4)	Reference			Reference		
Rural	27 (45.0)	134 (66.3)	28 (59.6)	0.63	0.33–1.41	0.065	**0.43**	**0.24–0.79**	**0.006**
**Educational level**									
None	8 (13.3)	15 (7.4)	0 (0.0)	Reference			Reference		
Primary	20 (33.3)	105 (52.0)	12 (25.5)	2.58	0.77–6.51	0.134	1.44	0.47–4.37	0.510
Secondary	21 (35.0)	65 (32.2)	27 (57.4)	**3.85**	**1.58–9.40**	**0.003**	1.81	0.56–5.82	0.313
Technical/professional	11 (18.3)	17 (8.4)	8 (17.0)	**2.25**	**1.09–6.71**	**0.030**	0.94	0.23–3.84	0.933
**Age at first intercourse (years)**									
<15	18 (30.0)	86 (42.6)	15 (31.9)	Reference			Reference		
16–20	17 (28.3)	61 (30.2)	15 (31.9)	1.21	0.73–2.01	0.155	1.19	0.99–2.02	0.050
≥21	13 (21.7)	35 (17.3)	9 (19.1)	2.81	0.82–9.06	0.098	**3.87**	**1.03–9.50**	**0.045**
**Lifetime amount of sexual partners**									
1	38 (63.3)	108 (53.5)	25 (53.2)	Reference			Reference		
2–3	13 (21.7)	40 (19.8)	14 (29.8)	1.50	0.82–2.75	0.184	1.58	0.80–3.11	0.183
>3	9 (15.0)	54 (26.7)	8 (17.0)	1.26	0.71–2.24	0.111	1.42	0.71–2.84	0.317
**Abortions**									
No	50 (83.3)	148 (73.3)	35 (74.5)	Reference			Reference		
Yes	10 (16.7)	54 (26.7)	12 (25.5)	1.47	0.86–2.52	0.158	1.69	0.92–3.13	0.090
**Condom use**									
Never	44 (73.3)	150 (74.3)	25 (53.2)	Reference			Reference		
Occasionally	9 (15.0)	35 (17.3)	15 (31.9)	1.59	0.69–3.68	0.272	1.80	0.99–3.74	0.050
Always	7 (11.7)	17 (8.4)	7 (14.9)	**2.09**	**1.11–3.94**	**0.021**	**3.11**	**1.16–8.33**	**0.023**
**Current smoker**									
No	49 (81.7)	184 (91.1)	45 (95.7)	Reference			Reference		
Yes	11 (18.3)	18 (8.9)	2 (4.3)	**0.41**	**0.18–0.94**	**0.035**	0.87	0.90–2.94	0.065

^a^ Estimates of logistic regression were adjusted (adjusted OR) for location of dwelling, educational level, age at first intercourse, lifetime amount of sexual partners, abortions, condom use and current smoking status.

OR, odds ratio; CI, confidence interval.

**Table 6:** Univariate and multivariate analysis of accessibility, perceived P&P programme quality and awareness of CC prevention mechanism associated with P&P programme attendance and compliance (n = 309 women).

Demographic	Total non- attendance	Attendance at least once	Attendance and compliance	Univariate analysis		*p*-value	Multivariate analysis		*p*-value
	*n* (%)	*n* (%)	*n* (%)	OR	95%CI		OR^a^	95%CI	
**Last P&P session held in your community**									
Less than 1 year	13 (44.8)	56 (41.2)	8 (28.6)	Reference			Reference		
More than 1 year	6 (20.7)	26 (19.1)	6 (21.4)	1.29	0.55–3.00	0.152	0.93	0.26–3.33	0.922
Never	10 (34.5)	54 (39.7)	14 (50.0)	1.58	0.80–3.15	0.185	1.01	0.39–2.58	0.973
**Time needed to reach P&P service centre**									
Less than 30 minutes	30 (50.0)	65 (32.2)	17 (36.2)	Reference			Reference		
30–60 minutes	10 (16.7)	57 (28.2)	12 (25.5)	0.90	0.55–1.47	0.690	3.79	0.60–9.63	0.153
More than 60 minutes	20 (33.3)	80 (39.6)	18 (38.3)	0.99	0.44–2.21	0.981	1.82	0.28–8.65	0.523
**Does your healthcentre/post have personnel who speak an indigenous language?**									
No	55 (91.7)	169 (83.7)	38 (80.9)	Reference			Reference		
Yes	5 (8.3)	33 (16.3)	9 (19.1)	1.68	0.89–3.17	0.107	1.01	0.29–3.52	0.986
**How long did it take to receive your cytology results?**									
1 to 15 days	0 --	53 (29.6)	17 (36.2)	Reference			Reference		
16 to 30 days	0 --	79 (44.1)	18 (38.3)	**0.49**	**0.27–0.88**	**0.018**	0.66	0.27–1.58	0.354
More than 30 days	0 --	47 (26.3)	12 (25.5)	0.63	0.32–1.22	0.175	0.54	0.19–1.53	0.248
**Do you know about CC?**									
No	19 (31.7)	69 (34.2)	7 (14.9)	Reference			Reference		
Yes	41 (68.3)	133 (65.8)	40 (85.1)	0.99	0.36–2.45	0.106	0.73	0.29–1.82	0.507
**Do you know about HPV infection?**									
No	32 (53.3)	136 67.3()	20 (42.6)	Reference			Reference		
Yes	28 (46.7)	66 (32.7)	27 (57.4)	1.20	0.74–1.93	0.145	1.84	0.69–4.89	0.219
**Do you know about the cytology examination?**									
No	0 --	7 (3.5)	5 (10.6)	Reference			Reference		
Yes	60 (100)	195 (96.5)	42 (89.4)	0.17	0.01–1.57	0.344	0.29	0.10–2.51	0.263
**Do you know when cytology examinations should begin?**									
No	19 (31.7)	74 (36.6)	7 (14.9)	Reference			Reference		
Yes	41 (68.3)	128 (63.4)	40 (85.1)	1.43	0.98–2.35	0.540	**2.69**	**1.08–6.68**	**0.032**
**Do you know the best way to diagnose CC?**									
No	35 (58.3)	104 (51.5)	12 (25.5)	Reference			Reference		
Yes	25 (41.7)	98 (48.5)	35 (74.5)	**2.13**	**1.30–3.47**	**0.002**	**2.43**	**1.02–5.77**	**0.043**
**Do you consider cytology important/useful?**									
No	30 (50.0)	65 (32.2)	8 (17.0)	Reference			Reference		
Yes	30 (50.0)	137 (67.8)	39 (83.0)	**2.65**	**1.59–4.41**	**0.001**	**2.64**	**1.12–6.19**	**0.025**

^a^ Estimates of logistic regression were adjusted (adjusted OR) for location of dwelling, last P&P session held in the community, time to reach P&P service, access, whether the healthcentre had personnel who could speak an indigenous language, how long it took to receive cytology results, is cytology important/useful and knowledge regarding CC, cytology examination, HPV and CC diagnosis methods.

OR, odds ratio; CI, confidence interval, P&P, promotion and prevention; CC, cervical cancer; Cvx, cervix; HPV, human papillomavirus.

## Discussion

The relevance of addressing CC-related matters in Latin America today concerns each country’s notorious variability regarding CC incidence and mortality rates, thereby highlighting differences in CC screening programme access and availability, as well as follow-up and treatment in cases where preneoplastic lesions have been detected [32]. Previous studies have indicated that the most affected populations are still the most demographically dispersed and socioeconomically marginalised and, therefore, the most neglected in terms of access to healthcare services and gynaecological care [33].

The Colombian National Health Survey estimated its childbearing age population’s sexual and reproductive health attitudes and practices; it reported that 12.7% of the women surveyed stated that they had never had a Papanicolaou test; however, variations among regions were observed as more than 20% of women from the Amazonas department stated that they had never had cytology during their lifetimes [11, 12], thereby agreeing with the present study’s data. The Amazon region’s panorama is similar to that in other Latin-American countries; a study in Mexico reported that 30% of sexually active women had never had cytology [34]. Another study in Rio Grande, Southern Brazil, stated that 57% of the 1,302 women interviewed reported never having had a Papanicolaou test [35].

The results indicated that even though close to 80% of the women included in this study had had cytology at some time, only 15% of them had adhered to the proposed CC P&P programme. Such results highlight a low CC programme attendance and compliance rate compared to those for other countries like France, where more than 80% of the women were up to date regarding CC detection [36], Canada where 70% of the women participated in routine CC detection screening and the United States where 85% of the women reported having had a pap test within the last 3 years [37].

Screening programme compliance in cities within countries bordering Colombia’s Amazon region has also been evaluated, indicating that although compliance rates are higher, regular attendance at healthcentre/posts for cytology is influenced by factors similar to those found in this study (Tables 5 and 6). This highlights the fact that factors related to geographical location and poor understanding of CC, its causes and prevention methods are associated with reduced P&P programme compliance [38–40].

Previous research has suggested that knowledge regarding a specific disease causes behavioural change tending towards prevention [23, 41]. This explains the positive associations within the multivariate model for most factors evaluated here within the knowledge dimension; it shows that women who had specific knowledge about the disease, prevention mechanisms, causal agent and diagnostic methods attended follow-up sessions on time (Table 6).

Some education-related factors were seen to be associated with the women in this study’s CC P&P programme attendance and compliance (Table 6); this suggested a greater probability of women having a higher educational level or some level of knowledge concerning HPV and CC prevention understanding Colombia’s health system and health service quality enforceability mechanisms [42]. These results were consistent with metanalysis results which identified women having the highest educational level being more likely to have been screened for CC. This suggested that such women had a higher probability (greater than 90%) of having had at least one Pap test over a three-year period compared to women having a lower educational level [43].

Broadening the target population’s level of knowledge and that of the health service staff caring for this population (scattered, vulnerable, rural, ethnically diverse) should contribute towards changing attitudes towards seeking consultation with healthcare providers and a decrease in erroneous concepts regarding CC P&P programmes [44–47].

Regular condom/contraceptive use (a relevant factor in multivariate analysis) was seen to be associated with P&P programme attendance and compliance. This might be explained by adherent women having more information regarding sexually-transmitted disease [48] or having access to health services arising from the need for information about planning methods; this would provide them with an opportunity to receive information from medical staff about the usefulness of cervical cytology and CC P&P programmes [34]. Having sufficient, pertinent, and timely information about CC and its conditioning factors would stimulate women’s desire for greater access to education and self-care [49, 50].

Another relevant factor in the multivariate model was better screening programme attendance and compliance in women who had begun their active sexual life after becoming 20 years of age. Previous reports have shown that age is associated with education levels, knowledge regarding CC and income. By contrast, young women and those beginning their sexual life at an early age represent priority population groups for orientating public policy regarding CC prevention and early detection as activities guaranteeing suitable screening programme attendance and compliance may not be sufficient for such population groups [51].

Geographical location was another related factor, as living in a rural environment or an area having high geographical dispersion could decrease screening programme attendance and compliance (i.e. displacement times required to reach programme sites) [9]. Previous research has shown that geographical dispersion [34, 52] is one of the barriers associated with poor access to cervical screening programmes in rural or remote populations, thereby leading to this population being involved in less participation [22]. This has also been associated with the lack of a family doctor, inconvenient clinic schedules, transportation problems, cultural barriers and indirect costs (childcare, taking time off work) [10]. Access factors representing possible associations with late diagnosis should be investigated in greater depth [53].

Cytology is a useful alternative for diagnosing abnormalities in regions having particular socio-environmental characteristics and difficult access. However, existing screening programmes should be rethought, taking the environment and its population’s characteristics into account (i.e. ethnicity, scattered population, availability of healthcare service providers); more relevant technologies for the Amazonian population should be included in P&P programmes (e.g. self-sampling for CC detection), mainly for those living in rural areas [8, 54].

P&P programme compliance-related factors described in other studies were not significant in this study (educational level, ethnic group, availability of people capable of translating Spanish into indigenous languages, distance to a healthcentre, age, monthly income and medical insurance) [53, 55–57]; however, some provided useful information for planning CC P&P programmes. A higher percentage of women in the total non-attendance group reported having no level of education, being affiliated to the subsidised health system, lacking monthly income and/or that a P&P programme had visited their community less than a year ago. Factors such as being dissatisfied with the P&P programme, taking more than 60 minutes to reach a P&P service centre and not knowing when cytology should begin were highlighted in the partial attendance and compliance group. A high percentage of women in the total attendance group stated that they had not received their last cytology results (Table 4, S2 and S3 Tables in S1 File).

CC prevention should be considered from a much broader perspective; intrapersonal, interpersonal, organisational, community and political characteristics should be considered to enable understanding the lack of P&P programme attendance and compliance and understanding if from points of view other than just a biomedical one. Many factors suggest the need for in-depth knowledge regarding other aspects (e.g. those related to organisation and administration) which also affect CC P&P programme operation. Understanding and making efforts to minimise all such barriers to access for identifying women at risk of developing cervical abnormalities will have an impact on timely CC diagnosis [55, 57, 58].

This study has reported P&P attendance- and compliance-related demographics and individual factors, many of which cannot be modified; however, behavioural models regarding health service access [59] highlight the influence of individual characteristics and the physical-social environment on health service use. Information obtained here might be useful for improving P&P programmes since they can be rethought using assertive and ethnically-socially relevant strategies.

World Health Organization guidelines for CC prevention-related screening and treatment of precancerous lesions recommend that HPV screening tests should be included in regions where P&P programmes are based on cytology followed by colposcopy and where indicators such as programme compliance are not adequately satisfied [60]. However, following such recommendations in environments such as Colombia’s Amazon region is excessively costly, wasteful and time-consuming. This means that visual inspection-based tests are useful because results concerning cervical architecture are immediate, thereby enabling preneoplastic lesions to be treated during the same visit. This obviously reduces difficulties regarding follow-up and contributes towards increasing CC screening programme attendance and compliance.

Latin America countries have made significant advances regarding cervical screening programmes’ coverage objectives. The challenge remains to ensure effective follow-up strategies for identifying and treating cervical lesions early and preventing them from progressing to CC [32]. It has been estimated that around 85% of the global CC burden occurs in developing countries and that 7.8% of female cancer-related deaths in South America are due to CC [32]. Only 69% of all women in Colombia diagnosed as having some type of cervical abnormality are detected in the disease’s early stages [14, 61].

The Amazon region’s specific characteristics highlight difficult access, high multidimensional poverty rates, low educational achievement, low per capita income, tenuous health care access and geographical dispersion. The Colombian Amazon region has one of Colombia’s highest mortality rates due to CC [19]. Studies regarding HPV distribution in this population have indicated greater than 60% viral prevalence and around 50% for simultaneous infections due to different types of HPV [62, 63]. CC screening programme coverage may have been improved but it is imperative that programmes should be properly introduced and run in rural and border areas.

## Limitations

The target population lived in scattered jungle/tropical forest communities having difficult access; this could obviously have affected target population selection and limited establishing the precise distance that women had to travel to the healthcare centre running the pertinent P&P programme, as well as other factors associated with cervical screening which have been reported in previous studies [22].

Local healthcare promoters were used for physically carrying out the surveys. Some answers to confidential questions may thus have been made in a guarded manner (i.e. answers given were convenient/expected/regarded as being most suitable). As periodic cervical cytology is socially expected behaviour it could have led to overestimation bias by the women being interviewed. Such limitations must be considered when designing future studies.

Self-reported data was used during data follow-up/analysis (i.e. depending on each woman’s memory); correct or erroneous answers may thus have been given (i.e. the body of information is considered susceptible to memory bias); future studies should therefore consider the women’s clinical records or, preferably, electronic medical records (EMR) as complementary tools when analysing CC screening programme attendance and compliance.

Although a significant amount of women participated in this study, future studies should include a larger population, especially studies carried out in jungle areas and those having greater dispersion. This should support ongoing results and provide information about other factors associated with CC P&P programme attendance and compliance (possibly those which were not detected in this study).

## Conclusions

The target population’s poor attendance at and compliance with CC screening programmes suggested that public health decision-makers should be actively involved in planning and running CC screening programmes through awareness-raising, educational strategies and considering the characteristics of the region’s population groups (e.g. educational level, ethnicity, access and native language use).

This study suggested that future public policy proposals should promote pertinent programmes guaranteeing that women’s geographical location should never be a barrier to receiving cytology examination, timely results and suitable follow-up per Colombian screening programmes’ 1–1–3 scheme.

Colombia’s Amazon region is a multi-ethnic, frontier region, having largescale population mobility, high poverty rates and limited access to health services. This study was the first one carried out in the region; it was aimed at evaluating factors having an impact on women’s attendance at and compliance with CC P&P programmes. The study contributes information referring to health service access within the framework of Universal Health Coverage (UHC) in heterogeneous settings having special characteristics. The factors involved in attendance and compliance consider regarding the perspectives of the women and healthcentre personnel involved and pose fresh challenges for approaching, introducing and running strategies for improving such programmes.

## Supporting information

S1 File Methodological guidelines for in-depth interviews and focus group discussions (FGDs). (DOCX)
