# Supplementary material for: Cervical cancer screening programme attendance and compliance predictors regarding Colombia’s Amazon region
Source: PLoS One. 2022 Jan 25;17(1):e0262069. doi: 10.1371/journal.pone.0262069 (PMC8789105; doi:10.1371/journal.pone.0262069)
Supplement: S1 File — (DOCX) [file pone.0262069.s001.docx]

**S1 File. Methodological guidelines for in-depth interviews and focus group discussions (FGDs)**

*In-depth interviews*: Methodological guidelines were developed for carrying out the in-depth interviews with health sector officials and indigenous and non-indigenous women during the FGDs in the Colombian municipalities of Leticia and Puerto Nariño. Methodological premises involved the researchers’ conceptual clarity concerning the purpose of the research (so as to be able to engage in satisfactory dialogue) and expression (because each session should offer different forms of expression for all individuals participating in an interview). The exchange of experiences and knowledge was encouraged in the interviews and FGDs. Knowing how to wait and listen was emphasised as qualitative research requires the sharing of experiences and concepts. Working intensely (but without creating tension) was encouraged as much more can be achieved in a meeting with smooth development of an interview/FGD conducted without exhausting the person being interviewed. A communicative atmosphere was encouraged as interviews/FGDs require a propitious atmosphere capable of providing spaces for communication. These were conducted by health sector officials involved in the Amazonas department’s promotion and prevention (P&P) programmes. A pre-test was carried out to determine the officials’ level of knowledge concerning the project, clarify doubts and present the project’s objective.

The following were considered selection criteria for participation in the in-depth interviews:

- Municipal and/or departmental health secretariat officials with some type of responsibility regarding cervical cancer (CC) surveillance and control, i.e. epidemiological, public health, sexual and/or reproductive surveillance
- Managers of subsidised and/or contributory healthcare insurance regime (HIR) insuring entities working in the municipalities of Leticia and Puerto Nariño;
- HIR staff responsible for CC surveillance and P&P programmes;
- Staff from Leticia and Puerto Nariño’s public and private networks healthcare service-providing institutions who were responsible for CC control (nurses, auxiliaries, gynaecologists, general practitioners, cytologists, pathologists); and
- Voluntarily accepting to participate in the study and signing an informed consent form.

The interviews that followed were based on the dimensions constructed from elements taken from the PRECEDE-PROCEED rural health planning model (predisposing, reinforcing and enabling causes in educational diagnosis and evaluation) [1] which has been used for designing healthcare P&P programmes aimed at behavioural change using information, education and communication (IEC) strategies [1, 2].

The questions posed during the interviews arose within the following dimensions:

- Socioeconomic: the municipality’s main economic activities, settlement patterns, social organization, expectations, desires and needs;
- Cultural–behavioural: cultural characterisation, most relevant cultural practices, cultural risk factors considered most relevant, relevant historical facts, migrations, potential, resistance to and capability for adapting to change and social support networks;
- Personal knowledge of the municipality’s ethnic diversity;
- Testimonies regarding previous experience aimed at improving women’s attendance and compliance with a particular P&P programme (local strategies);
- Previous experience of working with indigenous populations and healthcare P&P programmes;
- Epidemiological profile regarding health, demography, morbidity and mortality;
- Environmental: the municipality’s geographical characteristics and those of users attending the P&P programmes, access limitations, knowledge of users’ itinerary regarding access to healthcare services and the resources used for accessing healthcare services;
- Political–administrative: healthcare insurance, relationship with insurers and monitoring and control bodies, detailed description of functions, description of the network for providing healthcare services, characterising healthcare service use and testimonies regarding access to the CC screening/control programme;
- Description of P&P activities and how they are carried out;
- Users’ degree of satisfaction regarding healthcare-providing entities;
- Personal perception of P&P programme functioning;
- Level of knowledge and personal perception of current national regulations for controlling CC;
- Level of knowledge and perception of screening strategy quality; and
- Managing public health-related and epidemiological information.

*Focus group discussions (FGD)*: The invitation for women to participate in the focus groups was issued to the public and private sector institutions with which the project has an agreement (P&P programme users, user leagues and alliances, health promoters). A census of Leticia and Puerto Nariño’s institutions/actors was taken into account as well as of those carrying out activities in the neighbourhoods and communities forming part of the project’s study area: the Colombian Institute for Family Wellbeing/Welfare (ICBF), Social Action, universities, educational institutions, indigenous organisations, communities prioritised in the project (these communities were indicated by the *curacas,* who are considered the leaders of and spokesmen for the local indigenous people).

The following were considered the selection criteria for participating in the FDGs:

- Women aged 18 to 69 years old or young people under 18 years old leading an active sexual life (these age ranges were set in line with the Colombian Ministry of Social Protection’s guidelines for healthcare promotion and prevention regarding public health diseases and technical regulations for the early detection of CC and guidelines for dealing with preneoplastic lesions of the cervix) residing in Leticia and Puerto Nariño neighbourhoods, prioritised by the project [3];
- Women affiliated with the Colombian General Healthcare Social Security System (GHSSS);
- Women with some type of participation in or knowledge about early detection activities, procedures and interventions (i.e. CC screening), as established by Resolution 412/2000) [4];
- Indigenous women from communities having a distance of 1–20 km, 21–40 km, or more than 41 km to the closest healthcare centre (hospital/healthcare post) (these values were taken from scientific articles reviewing CC repercussions in patients having limited access to healthcare services) [5]; and
- Women who agreed to participate in the study and signed an informed consent form.

As with the interviews, a pre-test was carried out to investigate prior knowledge of the study’s objectives. Participants were then informed of the study’s proposed objectives and the methodology used for conducting the FGDs. Each participant’s voluntary decision to participate was verified and informed consent forms were signed.

The dimensions were constructed from elements taken from the PRECEDE-PROCEED rural health planning model [2] used for designing healthcare P&P programmes aimed at behavioural change using IEC strategies.

Focus groups were also orientated towards exploring predisposing factors, facilitators and reinforcers regarding each dimension. Five dimensions were addressed within the FGDs: socioeconomic and demographic factors, risk factors, accessibility to screening programmes, perceived CC screening programme quality and level of knowledge about CC prevention mechanisms, as stated below.

1. Socioeconomic and demographic factors: The survey included questions about age, type of access to the healthcare-related social security system, educational level, occupation during the past year, monthly income, ethnic group, public services provided and number of family members living in the home;
2. Risk factors: Questions regarding the number of children, number of sexual partners, age upon beginning sexual activity, planning method, condom use, smoking, whether the partner/husband supported participation in cytology, having had cytology at some point in life and lifetime number of cytology examinations;
3. P&P programme accessibility: The survey included questions about location of the home, means of transport used to reach the closest healthcare centre, time needed to travel from home to the healthcare centre, failing to attend a healthcare centre owing to excessive travel time, amount of money needed to attend a healthcare centre, the date on which the last healthcare day was held in the community, degree of satisfaction with healthcare days organised by the healthcare centre and whether personnel who spoke an indigenous language formed part of the P&P programmes;
4. Perceived CC screening programme quality: Questions were asked about the degree of satisfaction regarding the P&P programmes being offered, taking advantage of/seeking medical assistance in a country other than Colombia, aspects considered when seeking medical assistance in another country, access to results of the last cytology, medium used to deliver the cytology result, the reason why a result might not have been received, how long it took to receive the cytology result, perception of the time to access cytological results, understanding of cytological results and site where the cytology result was received; and
5. Level of knowledge about CC prevention mechanisms: The survey included questions about knowledge of CC, anatomical location of the cervix, knowledge about HPV, knowledge about the mode of HPV transmission, understanding the causes leading to development of CC, discerning CC identification methods, knowledge of cytology, knowledge of the appropriate age for beginning cytology, perception of cytology examination, knowledge of the benefits of cytology examination and the means through which knowledge of cytology can be acquired.

**S1 Table.** **Guiding questions asked during the in-depth interviews**

| **Main topic** | **Questions** |
| --- | --- |
| Socioeconomic | Where are you from?  What is your profession?  Tell me about your professional experience  How long have you been working at that institution?  How were the first years of work in the Amazonas department?  How do you feel now?  Which of personal values do you consider the most important?  What do you know about economic activities the department? |
| Cultural–behavioural | What do you know about the department?  What do you know about the culture, beliefs, and Amazonian people’s ways of life?  What do you know about the indigenous people?  What part of the department's history would you like to tell?  In your opinion, what are the main values of Amazonian people? |
| Epidemiological | In your knowledge, what are the public health problems that affect the population of the department/municipality?  What do you know about the sexual and reproductive health profile of the people living in the department/municipality?  Do you know what is the first cause of death in women and men in the department/ municipality?  Do you know which is the first cause of death by cancer in women from the department? |
| Environmental | How is the healthcare system ​in the department?  Which health-promoting companies (HPC) and healthcare-providing institutions (HPI) are working in the department/municipality?  How often are extramural health campaigns being carried out in urban and rural areas?  What do you know about the CC P&P programmes running in the department?  What is your perception of CC P&P programme quality and opportunity?  What is your perception about access to cervical cytology and its quality and promptness?  What is your perception of the health personnel responsible for CC P&P programmes?  Which characteristics/attitudes have you identified that could facilitate women’s attendance at and compliance with CC P&P programmes?  Which characteristics/attitudes have you identified that could hamper women’s attendance at and compliance with CC P&P programmes? |
| Political/policy-based–administrative | What is your perception regarding the officials/administrative staff managing CC P&P programmes?  How do you think attendance at and compliance with CC P&P programmes could be improved?  What role do users have in the healthcare system and regarding CC P&P programme service provision quality?  What roles do HPC and HPI play regarding CC P&P programme service provision quality?  What do you know about legislation and Colombia’s technical standards regarding CC P&P programmes in the department/municipality? |

**S2 Table.** **Univariate analysis of socioeconomic and demographic and risk factors associated with attendance and compliance** **among women included in the study (n=309)**

| **Demographics** | **Total non- attendance** | | **Attendance at least once** | | **Attendance and compliance** | | **Univariate**  **analysis** | | ***p-*value** |
| --- | --- | --- | --- | --- | --- | --- | --- | --- | --- |
|  | **n** | **(%)** | **n** | **(%)** | **n** | **(%)** | **OR** | **95%CI** |  |
| **Age (years)** |  |  |  |  |  |  |  |  |  |
| 18–34 | 25 | (41.7) | 87 | (43.1) | 24 | (51.1) | Reference | |  |
| 35–50 | 20 | (33.3) | 91 | (45.0) | 14 | (29.8) | 0.86 | 0.52–1.44 | 0.587 |
| >50 | 15 | (25.0) | 24 | (11.9) | 9 | (19.1) | 0.69 | 0.33–1.41 | 0.315 |
| **Ethnicity** |  |  |  |  |  |  |  |  |  |
| Ticuna | 29 | (48.3) | 125 | (61.9) | 26 | (55.3) | Reference | |  |
| Mestizo | 22 | (36.7) | 39 | (19.3) | 17 | (36.2) | 0.75 | 0.39–1.41 | 0.380 |
| Other indigenous ^a^ | 9 | (15.0) | 36 | (17.8) | 4 | (8.5) | 0.78 | 0.44–1.37 | 0.398 |
| Afro descendent | 0 | -- | 1 | (0.5) | 1 | (2.1) | 5.55 | 0.37–9.98 | 0.213 |
| **Location of dwelling** |  |  |  |  |  |  |  |  |  |
| Urban | 33 | (55.0) | 68 | (33.7) | 19 | (40.4) | Reference | |  |
| Rural | 27 | (45.0) | 134 | (66.3) | 28 | (59.6) | 0.63 0.39–1.02 | | 0.065 |
| **Educational level** |  |  |  |  |  |  |  |  |  |
| None | 8 | (13.3) | 15 | (7.4) | 0 | (0.0) | Reference | |  |
| Primary | 20 | (33.3) | 105 | (52.0) | 12 | (25.5) | 2.58 | 0.77–6.51 | 0.134 |
| Secondary | 21 | (35.0) | 65 | (32.2) | 27 | (57.4) | **3.85** | **1.58–9.40** | **0.003** |
| Technical/Professional | 11 | (18.3) | 17 | (8.4) | 8 | (17.0) | **2.25** | **1.09–6.71** | **0.030** |
| **Occupation** |  |  |  |  |  |  |  |  |  |
| Homemaker | 21 | (35.0) | 86 | (42.6) | 14 | (29.8) | Reference | |  |
| Agriculture-related | 16 | (26.7) | 61 | (30.2) | 15 | (31.9) | 1.00 | 0.37–2.73 | 0.986 |
| Businesswoman | 4 | (6.7) | 12 | (5.9) | 3 | (6.4) | 1.17 | 0.67–2.03 | 0.579 |
| Other | 19 | (31.7) | 43 | (21.3) | 15 | (31.9) | 1.00 | 0.55–1.81 | 0.993 |
| **Healthcare scheme affiliation** |  |  |  |  |  |  |  |  |  |
| Subsidized-linked | 51 | (85.0) | 148 | (73.3) | 39 | (83.0) | Reference | |  |
| Contributory/private | 9 | (15.0) | 54 | (26.7) | 8 | (17.0) | 0.96 | 0.51–1.82 | 0.567 |
| **Average monthly income ^b^** |  |  |  |  |  |  |  |  |  |
| No earnings | 14 | (23.3) | 43 | (21.3) | 7 | (14.9) | Reference | |  |
| <Minimum | 28 | (46.7) | 131 | (64.9) | 23 | (48.9) | 1.32 | 0.74–2.36 | 0.337 |
| Minimum | 5 | (8.3) | 15 | (7.4) | 7 | (14.9) | 1.45 | 0.48–4.37 | 0.503 |
| ≥Minimum | 13 | (21.7) | 13 | (6.4) | 10 | (21.3) | 1.02 | 0.42–2.48 | 0.958 |
| **Access to public services** |  |  |  |  |  |  |  |  |  |
| None | 4 | (6.7) | 22 | (10.9) | 1 | (2.1) | Reference | |  |
| Electricity | 49 | (81.7) | 148 | 73.3) | 37 | (78.7) | 0.94 | 0.42–2.10 | 0.885 |
| Water/sewerage | 7 | (11.7) | 32 | (15.8) | 9 | (19.1) | 1.31 | 0.50–3.42 | 0.578 |
| **Age at first intercourse (years)** |  |  |  |  |  |  |  |  |  |
| <15 | 18 | (30.0) | 86 | (42.6) | 15 | (31.9) | Reference | |  |
| 16–20 | 17 | (28.3) | 61 | (30.2) | 15 | (31.9) | 1.21 | 0.73–2.01 | 0.155 |
| ≥21 | 13 | (21.7) | 35 | (17.3) | 9 | (19.1) | 2.81 | 0.82–9.06 | 0.098 |
| **Lifetime number of sexual partners** |  |  |  |  |  |  |  |  |  |
| 1 | 38 | (63.3) | 108 | (53.5) | 25 | (53.2) | Reference | |  |
| 2–3 | 13 | (21.7) | 40 | (19.8) | 14 | (29.8) | 1.50 | 0.82–2.75 | 0.184 |
| >3 | 9 | (15.0) | 54 | (26.7) | 8 | (17.0) | 1.26 | 0.71–2.24 | 0.111 |
| **Pregnancies** |  |  |  |  |  |  |  |  |  |
| None | 2 | (3.3) | 4 | (2.0) | 0 | -- | Reference | |  |
| 1–2 | 17 | (28.3) | 42 | (20.8) | 22 | (46.8) | 3.74 | 0.40–8.27 | 0.242 |
| ≥3 | 41 | (68.3) | 156 | (77.2) | 25 | (53.2) | 2.30 | 0.26–9.28 | 0.452 |
| **Abortions** |  |  |  |  |  |  |  |  |  |
| No | 50 | (83.3) | 148 | (73.3) | 35 | (74.5) | Reference | |  |
| Yes | 10 | (16.7) | 54 | (26.7) | 12 | (25.5) | 1.47 | 0.86–2.52 | 0.158 |
| **Contraceptive method** |  |  |  |  |  |  |  |  |  |
| None | 18 | (30.0) | 86 | (42.6) | 15 | (31.9) | Reference | |  |
| Hormonal | 17 | (28.3) | 61 | (30.2) | 15 | (31.9) | 1.04 | 0.59–1.81 | 0.887 |
| Tubal ligation | 13 | (21.7) | 35 | (17.3) | 9 | (19.1) | 0.86 | 0.45–1.67 | 0.675 |
| Other methods ^c^ | 12 | (20.0) | 19 | (9.4) | 9 | (19.1) | 0.92 | 0.42–1.98 | 0.833 |
| **Condom/contraceptive use** |  |  |  |  |  |  |  |  |  |
| Never | 44 | (73.3) | 150 | (74.3) | 25 | (53.2) | Reference | |  |
| Occasionally | 9 | (15.0) | 35 | (17.3) | 15 | (31.9) | 1.59 | 0.69–3.68 | 0.272 |
| Always | 7 | (11.7) | 17 | (8.4) | 7 | (14.9) | **2.09** | **1.11–3.94** | **0.021** |
| **Current smoker** |  |  |  |  |  |  |  |  |  |
| No | 49 | (81.7) | 184 | (91.1) | 45 | (95.7) | Reference | |  |
| Yes | 11 | (18.3) | 18 | (8.9) | 2 | (4.3) | **0.41** | **0.18–0.94** | **0.035** |

OR, odds ratio; CI, confidence interval.

^a^ Other indigenous group included Uitoto, Yagua and Cocama.

^b^ Minimum average monthly income (2016 rate) equal to roughly USD 240.

**^c^** Other methods include medicinal plants and traditional means of contraception.

**S3 Table.** **Univariate analysis of P&P programme accessibility, perceived P&P quality and awareness of CC prevention mechanisms associated with attendance and compliance** **among women included in the study (n=309)**

| **Demographic** | **Total non- attendance** | | **Attendance at least once** | | **Attendance and compliance** | | **Univariate**  **analysis** | |  |
| --- | --- | --- | --- | --- | --- | --- | --- | --- | --- |
|  | **n** | **(%)** | **n** | **(%)** | **n** | **(%)** | **OR** | **95%CI** | ***p-*values** |
| **Last P&P session in your community** |  |  |  |  |  |  |  |  |  |
| Less than 1 year ago | 13 | (44.8) | 56 | (41.2) | 8 | (28.6) | Reference | |  |
| More than 1 year ago | 6 | (20.7) | 26 | (19.1) | 6 | (21.4) | 1.29 | 0.55–3.00 | 0.152 |
| Never | 10 | (34.5) | 54 | (39.7) | 14 | (50.0) | 1.58 | 0.80–3.15 | 0.185 |
| **Means of transport for attending P&P** |  |  |  |  |  |  |  |  |  |
| Boat/canoe | 27 | (45.0) | 109 | (54.0) | 22 | (46.8) | Reference | |  |
| Motorbike/car | 31 | (51.7) | 70 | (34.7) | 24 | (51.1) | 0.90 | 0.55–1.47 | 0.690 |
| Walking | 2 | (3.3) | 23 | (11.4) | 1 | (2.1) | 0.99 | 0.44–2.21 | 0.981 |
| **Time needed to reach P&P service centre** |  |  |  |  |  |  |  |  |  |
| Less than 30 minutes | 30 | (50.0) | 65 | (32.2) | 17 | (36.2) | Reference | |  |
| 30–60 minutes | 10 | (16.7) | 57 | (28.2) | 12 | (25.5) | 1.67 | 0.92–3.04 | 0.090 |
| More than 60 minutes | 20 | (33.3) | 80 | (39.6) | 18 | (38.3) | 1.40 | 0.81–2.41 | 0.200 |
| **Did you receive the result of your last cytology?** |  |  |  |  |  |  |  |  |  |
| No | 0 | -- | 175 | (86.6) | 44 | (93.6) | Reference | |  |
| Yes | 0 | -- | 27 | (13.4) | 3 | (6.4) | 1.34 | 0.64–2.78 | 0.426 |
| **Where did you receive your cytology result?** |  |  |  |  |  |  |  |  |  |
| I did not collect it | 0 | -- | 16 | (7.9) | 2 | (4.3) | Reference | |  |
| Hospital or clinic | 0 | -- | 124 | (61.4) | 35 | (74.5) | 1.25 | 0.50–3.12 | 0.631 |
| In your home/community | 0 | -- | 62 | (30.7) | 10 | (21.3) | 0.71 | 0.27–1.86 | 0.492 |
| **How much does it cost to travel to a health centre running a P&P programme?** |  |  |  |  |  |  |  |  |  |
| ≤5 USD | 32 | (53.3) | 91 | (45.0) | 23 | (48.9) | Reference | |  |
| 6–10 USD | 15 | (25.0) | 53 | (26.2) | 14 | (29.8) | 1.24 | 0.71–2.19 | 0.440 |
| >10 USD | 13 | (21.7) | 58 | (28.7) | 10 | (21.3) | 1.14 | 0.65–2.00 | 0.635 |
| **Does your health centre have personnel who can speak an indigenous language?** |  |  |  |  |  |  |  |  |  |
| No | 55 | (91.7) | 169 | (83.7) | 38 | (80.9) | Reference | |  |
| Yes | 5 | (8.3) | 33 | (16.3) | 9 | (19.1) | 1.68 | 0.89–3.17 | 0.107 |
| **Have you been seen by a doctor in another country?** |  |  |  |  |  |  |  |  |  |
| No | 4 | (6.7) | 7 | (3.5) | 6 | (12.8) | Reference | |  |
| Yes | 56 | (93.3) | 195 | (96.5) | 41 | (87.2) | 0.64 | 0.21–1.96 | 0.443 |
| **Does the distance to a health centre limit your attending a P&P programme?** |  |  |  |  |  |  |  |  |  |
| No | 37 | (61.7) | 114 | (56.4) | 27 | (57.4) | Reference | |  |
| Yes | 23 | (38.3) | 88 | (43.6) | 20 | (42.6) | 1.09 | 0.68–1.74 | 0.697 |
| **How long did it take for you to receive your cytology results?** |  |  |  |  |  |  |  |  |  |
| 1 to 15 days | 0 | -- | 53 | (29.6) | 17 | (36.2) | Reference | |  |
| 16 to 30 days | 0 | -- | 79 | (44.1) | 18 | (38.3) | **0.49** | **0.27–0.88** | **0.018** |
| More than 30 days | 0 | -- | 47 | (26.3) | 12 | (25.5) | 0.63 | 0.32–1.22 | 0.175 |
| **How do you rate cytology result delivery times?** |  |  |  |  |  |  |  |  |  |
| Appropriate | 0 | -- | 83 | (41.1) | 17 | (36.2) | Reference | |  |
| Not appropriate | 0 | -- | 119 | (58.9) | 30 | (63.8) | 0.83 | 0.51–1.35 | 0.469 |
| **Who read your last cytology result?** |  |  |  |  |  |  |  |  |  |
| Another person | 0 | -- | 6 | (3.0) | 0 | -- | Reference | |  |
| A nurse/doctor | 0 | -- | 176 | (87.1) | 45 | (95.7) | 1.46 | 0.31–6.87 | 0.627 |
| A healthcare promoter | 0 | -- | 20 | (9.9) | 2 | (4.3) | 1.28 | 0.23–7.11 | 0.773 |
| **Did you understand the cytology result?** |  |  |  |  |  |  |  |  |  |
| No | 0 | -- | 103 | (51.0) | 19 | (40.4) | Reference | |  |
| Yes | 0 | -- | 99 | (49.0) | 28 | (59.6) | 1.24 | 0.77–1.99 | 0.367 |
| **Do you know what to do after receiving a cytology result?** |  |  |  |  |  |  |  |  |  |
| No | 9 | (15.0) | 42 | (20.8) | 6 | (12.8) | Reference | |  |
| Yes | 51 | (85.0) | 160 | (79.2) | 41 | (87.2) | 0.98 | 0.54–1.77 | 0.951 |
| **Are you satisfied with the P&P programme?** |  |  |  |  |  |  |  |  |  |
| Unsatisfied | 18 | (30.0) | 66 | (64.7) | 18 | (38.3) | Reference | |  |
| Satisfied | 42 | (70.0) | 36 | (35.3) | 29 | (61.7) | 0.78 | 0.48–1.28 | 0.340 |
| **Does your partner agree with cytology?** |  |  |  |  |  |  |  |  |  |
| No | 10 | (18.5) | 20 | (11.0) | 5 | (12.8) | Reference | |  |
| Yes | 44 | (81.5) | 162 | (89.0) | 34 | (87.2) | 1.48 | 0.69–3.08 | 0.310 |
| **Do you know about CC?** |  |  |  |  |  |  |  |  |  |
| No | 19 | (31.7) | 69 | (34.2) | 7 | (14.9) | Reference | |  |
| Yes | 41 | (68.3) | 133 | (65.8) | 40 | (85.1) | 0.99 | 0.36–2.45 | 0.106 |
| **Do you know where your cervix is in your body?** |  |  |  |  |  |  |  |  |  |
| No | 16 | (26.7) | 60 | (29.7) | 6 | (12.8) | Reference | |  |
| Yes | 44 | (73.3) | 142 | (70.3) | 41 | (87.2) | 1.43 | 0.85–2.38 | 0.257 |
| **Do you know about HPV infection?** |  |  |  |  |  |  |  |  |  |
| No | 32 | (53.3) | 136 | 67.3() | 20 | (42.6) | Reference | |  |
| Yes | 28 | (46.7) | 66 | (32.7) | 27 | (57.4) | 1.20 | 0.74–1.93 | 0.145 |
| **Do you know that HPV can be sexually transmitted?** |  |  |  |  |  |  |  |  |  |
| No | 36 | (60.0) | 149 | (73.8) | 26 | (55.3) | Reference | |  |
| Yes | 24 | (40.0) | 53 | (26.2) | 21 | (44.7) | 1.37 | 0.70–2.65 | 0.349 |
| **Do you know about the cytology examination?** |  |  |  |  |  |  |  |  |  |
| No | 0 | -- | 7 | (3.5) | 5 | (10.6) | Reference | |  |
| Yes | 60 | (100) | 195 | (96.5) | 42 | (89.4) | 0.17 | 0.01–1.57 | 0.344 |
| **Do you know when cytology should begin?** |  |  |  |  |  |  |  |  |  |
| No | 19 | (31.7) | 74 | (36.6) | 7 | (14.9) | Reference | |  |
| Yes | 41 | (68.3) | 128 | (63.4) | 40 | (85.1) | 1.43 | 0.98–2.35 | 0.540 |
| **Do you know the best way to diagnose CC?** |  |  |  |  |  |  |  |  |  |
| No | 35 | (58.3) | 104 | (51.5) | 12 | (25.5) | Reference | |  |
| Yes | 25 | (41.7) | 98 | (48.5) | 35 | (74.5) | **2.13** | **1.30–3.47** | **0.002** |
| **Do you consider cytology important/useful?** |  |  |  |  |  |  |  |  |  |
| No | 30 | (50.0) | 65 | (32.2) | 8 | (17.0) | Reference | |  |
| Yes | 30 | (50.0) | 137 | (67.8) | 39 | (83.0) | **2.65** | **1.59–4.41** | **0.001** |
| **What could influence (your) participation in a P&P programme?** |  |  |  |  |  |  |  |  |  |
| Own initiative | 50 | (83.3) | 145 | (71.8) | 37 | (78.7) | Reference | |  |
| Healthcare/service personnel | 4 | (6.7) | 34 | (16.8) | 5 | (10.6) | 1.22 | 0.63–2.36 | 0.552 |
| Family members/friends | 6 | (10.0) | 23 | (11.4) | 5 | (10.6) | 1.10 | 0.52–2.30 | 0.794 |

OR, odds ratio; CI, confidence interval.

**S4 Table.** **Power analysis regarding socioeconomic, demographic and risk factors included in multivariate analysis**

| **Demographics** | **Multivariate analysis** | | | |  |
| --- | --- | --- | --- | --- | --- |
|  | **OR**^a^ | **95%CI** | ***p-*value** | **Power analysis** |  |
| **Location of dwelling** |  |  |  |  |  |
| Urban | Reference | |  |  |  |
| Rural | **0.43** | **0.24-0.79** | **0.006** | **0.949** |  |
| **Educational level** |  |  |  |  |  |
| None | Reference | |  |  |  |
| Primary | 1.44 | 0.47–4.37 | 0.510 | 0.342 |  |
| Secondary | 1.81 | 0.56–5.82 | 0.313 | 0.714 |  |
| Technical/professional | 0.94 | 0.23–3.84 | 0.933 | 0.045 |  |
| **Age at first intercourse (years)** |  |  |  |  |  |
| <15 | Reference | |  |  |  |
| 16–20 | 1.19 | 0.99–2.02 | 0.050 | 0.111 |  |
| ≥21 | **3.87** | **1.03–9.50** | **0.045** | **0.999** |  |
| **Lifetime amount of sexual partners** |  |  |  |  |  |
| 1 | Reference | |  |  |  |
| 2–3 | 1.58 | 0.80–3.11 | 0.183 | 0.495 |  |
| >3 | 1.42 | 0.71–2.84 | 0.317 | 0.320 |  |
| **Abortions** |  |  |  |  |  |
| No | Reference | |  |  |  |
| Yes | 1.69 | 0.92–3.13 | 0.090 | 0.608 |  |
| **Condom use** |  |  |  |  |  |
| Never | Reference | |  |  |  |
| Occasionally | 1.80 | 0.99–3.74 | 0.050 | 0.706 |  |
| Always | **3.11** | **1.16–8.33** | **0.023** | **0.998** |  |
| **Current smoker** |  |  |  |  |  |
| No | Reference | |  |  |  |
| Yes | 0.87 | 0.90–2.94 | 0.065 | 0.992 |  |

**S5 Table.** **Power analysis for P&P programme accessibility, perceived P&P quality and awareness of CC prevention mechanisms included in multivariate analysis**

| **Demographic** | **Multivariate analysis** | | | |
| --- | --- | --- | --- | --- |
|  | **OR**^a^ | **95%CI** | ***p-*value** | **Power analysis** |
| **Last P&P session held in your community** |  |  |  |  |
| Less than 1 year | Reference | |  |  |
| More than 1 year | 0.93 | 0.26–3.33 | 0.922 | 0.043 |
| Never | 1.01 | 0.39–2.58 | 0.973 | 0.027 |
| **Time needed to reach P&P service centre** |  |  |  |  |
| Less than 30 minutes | Reference | |  |  |
| 30–60 minutes | 3.79 | 0.60–9.63 | 0.153 | 0.999 |
| More than 60 minutes | 1.82 | 0.28–8.65 | 0.523 | 0.722 |
| **Does your healthcentre/post have personnel who speak an indigenous language?** |  |  |  |  |
| No | Reference | |  |  |
| Yes | 1.01 | 0.29–3.52 | 0.986 | 0.028 |
| **How long did it take to receive your cytology results?** |  |  |  |  |
| 1 to 15 days | Reference | |  |  |
| 16 to 30 days | 0.66 | 0.27–1.58 | 0.354 | 0.327 |
| More than 30 days | 0.54 | 0.19–1.53 | 0.248 | 0.610 |
| **Do you know about CC?** |  |  |  |  |
| No | Reference | |  |  |
| Yes | 0.73 | 0.29–1.82 | 0.507 | 0.268 |
| **Do you know about HPV infection?** |  |  |  |  |
| No | Reference | |  |  |
| Yes | 1.84 | 0.69–4.89 | 0.219 | 0.737 |
| **Do you know about the cytology examination?** |  |  |  |  |
| No | Reference | |  |  |
| Yes | 0.29 | 0.10–2.51 | 0.263 | 0.990 |
| **Do you know when cytology examinations should begin?** |  |  |  |  |
| No | Reference | |  |  |
| Yes | **2.69** | **1.08–6.68** | **0.032** | **0.988** |
| **Do you know the best way to diagnose CC?** |  |  |  |  |
| No | Reference | |  |  |
| Yes | **2.43** | **1.02–5.77** | **0.043** | **0.966** |
| **Do you consider cytology important/useful?** |  |  |  |  |
| No | Reference | |  |  |
| Yes | **2.64** | **1.12–6.19** | **0.025** | **0.985** |

**References**

1. Tramm R, McCarthy A, Yates P. Using the Precede-Proceed Model of Health Program Planning in breast cancer nursing research. J Adv Nurs. 2012;68(8):1870-80. <https://www.ncbi.nlm.nih.gov/pubmed/22142482> PMID: 22142482

2. Green LW, Kreuter MW. Health promotion planning: an eductional and ecological approach. 4th ed. . New York: McGraw-Hill; 2005.

3. MinSalud. Ministerio de la Protección Social. Norma Técnica para la Detección Temprana del Cancer Cuello Uterino y Guía de Atención de Lesiones Preneoplásicas de Cuello Uterino. 2014 <http://gpc.minsalud.gov.co/gpc_sites/Repositorio/Otros_conv/GPC_Cuello_Uterino/gpc_c_uterino.aspx> (Accessed 6 Dec 2018).

4. Resolución 412. Por la cual se establecen las actividades, procedimientos e intervenciones de demanda inducida y obligatorio cumplimiento y se adoptan las normas técnicas y guías de atención para el desarrollo de las acciones de protección específica y detección temprana y la atención de enfermedades de interés en salud pública. 2000 <https://docs.supersalud.gov.co/PortalWeb/Juridica/OtraNormativa/R0412000.pdf> (Accessed 6 Dec 2018).

5. Terán Y, Carreón P, Moya M, Cuevas S, Range N, Gutiérrez S, et al. Repercusiones del cáncer cervicouterino en pacientes con limitaciones de acceso a los servicios de salud. Ginecol Obstet Mex 2015;8:162-72. <https://ginecologiayobstetricia.org.mx/secciones/articulos-originales-numero83/repercusion-del-cancer-cervicouterino-en-pacientes-con-limitaciones-de-acceso-a-los-servicios-de-salud/>
